# Accuracy of Pedicle Screw Placement Using Intraoperative CT-Guided Navigation and Conventional Fluoroscopy for Lumbar Spondylosis

**DOI:** 10.7759/cureus.17431

**Published:** 2021-08-25

**Authors:** Ashwaq Alqurashi, Soha A Alomar, Mohamad Bakhaidar, Mohammed Alfiky, Saleh S Baeesa

**Affiliations:** 1 Department of Surgery, King Abdulaziz University Hospital, Jeddah, SAU; 2 Department of Surgery, Faculty of Medicine, King Abdulaziz University, Jeddah, SAU; 3 Division of Neurosurgery, Department of Surgery, Faculty of Medicine, King Abdulaziz University, Jeddah, SAU

**Keywords:** spine, intraoperative ct, navigation, conventional fluoroscopy, pedicle screws accuracy

## Abstract

Background

Transpedicular screws are a common adjunct for lumbar spine fusion. Accurate screw placement to prevent neurological injury has been the subject of many studies. The adoption of spine neuronavigation has shown a significant decrease in screw malposition morbidity. We aim to evaluate the accuracy of pedicle screw insertion using intraoperative CT-guided navigation in lumbar spondylosis.

Methods

We reviewed a prospective registry-based cohort study. This included patients who underwent transpedicular screws insertion for lumbar spondylosis under intraoperative CT-guided navigation (iCT-Nav) and compared it to another group operated using conventional fluoroscopy (FS) over one year. In addition, the correlation between clinical outcome using the visual analog scale (VAS) and short 12 physical component scores (SF-12 PCS) and hospital stay was reported.

Results

Fifteen patients were included in the iCT-Nav group compared to 42 patients in the FS group. The median age of the iCT-Nav group was 59.3 years old (27-76 years) versus 45 years old (20-60 years) in the FS group. The number of screws was 98 in the iCT-Nav group and 252 screws in the FS group. Based on more than 2-mm breach increments measured on CT images, lumbar pedicular screw placement accuracy was 100% in the iCT-Nav group and 86.9% in the FS group. None of the patients in the iCT-Nav group had to undergo any postoperative revisions. On the other hand, two patients of the FS group developed new postoperative symptoms related to displaced screws and required readmission and revision surgery.

Conclusion

In a commonly performed pedicular fixation in lumbar spondylosis, iCT-Nav has been shown to improve the accuracy of pedicle screw placement, hospital stay, and functional outcomes compared to FS.

## Introduction

Since King's introduction of pedicle screws in 1944, transpedicular screws (TPS) have emerged as an essential tool for spinal fusion for various clinical indications [1]. Degenerative disc disease, spondylolysis and spondylolisthesis, fractured vertebrae, spondylitis, and vertebral tumors are common indications of spinal fusion. This procedure may carry a risk of screw misplacement, with a possibility of neural damage, particularly if the medial cortex of the pedicle were penetrated [2]. The risk of causing a new neurological pain or deficit is considered while doing this procedure and addressed with patients before surgery. Advancements in imaging have led to the use of intraoperative fluoroscopy to promote accuracy in screw placement. This method is associated with increased radiation exposure to the patient and surgeon and increased operative time [3]. Accuracy of pedicular screws placement has been the subject of many studies, in which a wide range of screw malposition rates has been reported around 1.5-29% [2-4].

We present our experience by comparing the outcome and accuracy of screw placement in patients who underwent a lumbar fusion for degenerative lumbar disk disease using the conventional fluoroscopy method to intraoperative CT-guided navigation.

## Materials and methods

The study was approved by the Faculty of Medicine, King Abdulaziz University Research Ethical Committee (No. 289-17).

Study design

We have conducted a prospective registry-based cohort study with a total of 57 patients who had single or multiple-level surgery for degenerative lumbar spondylosis between March 2016 and April 2017 in the Division of Neurosurgery at King Abdulaziz University Hospital, Jeddah, Saudi Arabia. We included all patients who underwent an instrumented lumbar fusion using transpedicular screws under an intraoperative CT-guided navigation system and conventional fluoroscopy during the same period. We excluded children younger than 15 years old and adult patients with previous spine surgery, deformity, or lumbar spine surgery for reasons other than spondylosis. In addition, patients who had cervical or thoracic pedicular screws were not included. According to the intraoperative screw placement image-guided methods, the patients were divided in a nonrandomized way into two following groups: intraoperative CT-guided navigation (iCT-Nav) and conventional fluoroscopy (FS). They had at least two years of follow-up. 

Data collection

The analyzed demographics and preoperative data included age, gender, and body mass index (BMI). The operative and perioperative collected data included total screws used, operative time, surgical-related complications, and hospitalization days. Also, visual analog scale (VAS, scale 0-10) and short 12 physical component scores (SF-12 PCS) were assessed. The follow-up time was scheduled at the preoperative visit, postoperative six weeks, six months, one year, and two years as per the setup by the prospective registry. 

Surgical technique

**Figure 1:**
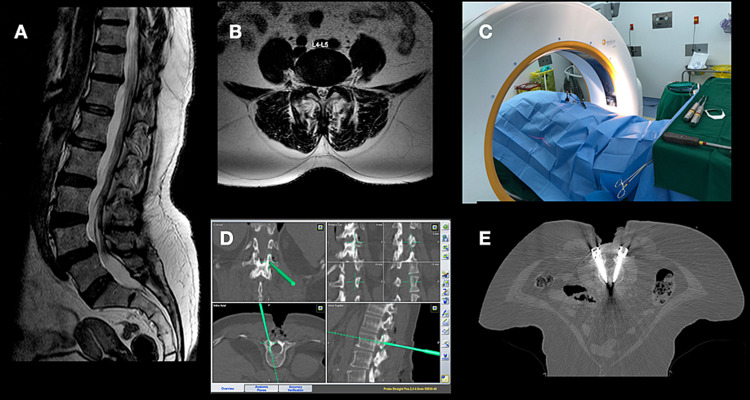
Case illustration of a 55-year-old female presented with low back pain and bilateral radiculopathy due to L4-5 spondylolisthesis as demonstrated in the sagittal (A) and axial T2-weighted MRI. Intraoperative image acquisition was obtained (C) using iCT-Nav, and TPS placement was performed (D). Screws placements were checked (E), and then the procedure was completed with underwent decompression and fusion. iCT-Nav: intraoperative CT-guided navigation; TPS: transpedicular screws

All patients had conventional open surgery by one senior surgeon. The insertion of TPS in the iCT-Nav group was facilitated using a 40-slice CT scanner (Airo, BrainLab AG, Germany) and a frameless infrared-based navigation system (VectorVision, BrainLab AG, Germany). After adequate localization and exploring the spinal levels, a reference frame was securely attached to the spinous process of the planned lowermost instrumented vertebra for registration. Then a control CT scan was performed. Images were retrieved and transferred from the CT scanner to the navigation workstation to produce a 3D image to provide automatic registration (Figure 1). After verifying the registration of the target vertebrae, the pedicle entry point and the trajectory were located, and the length and diameter of each screw using the registered pointer were displayed through sagittal and axial views. A freehand pilot hole was then prepared with the drill guide using preregistered tools with the assistance of a navigation system. A CT confirmation scan was immediately performed following all TPS placement. After dissection and retraction of paravertebral muscles in the FS group, a free hand intersection technique was used to insert the screws according to the anatomical landmarks with lateral C-arm fluoroscopy guidance (Siemens AG, Munich, Germany). After screws placement, two fluoroscopic views were obtained for confirmation.

Evaluation of screw placement

The screw positions were evaluated using intraoperative acquired CT images in the iCT-Nav group and postoperative CT images for the FS group. The screws' positions were assessed and classified according to the classification systems introduced by Gertzbein and Robbins, with a 2-mm increment to evaluate the severity of screw malposition [5]. The classification system is as follows: (a) TPS without breach of the cortical layer of the pedicle, (b) the minor breaching of cortex with less than 2 mm of displacement, (c) moderate displacement of 2-4 mm, and (d) severe displacement >4 mm [5]. The evaluation was performed by two surgeons with a 0.80 kappa coefficient for inter-observer reliability.

Statistical analysis

The collected data were analyzed using SPSS statistical program version 20 (Chicago, IL: SPSS Inc.). Fisher's exact test has been used to compare categorical variables. P-value <0.05 is considered statistically significant.

## Results

Demographic

During the study period, 57 patients were included, 15 patients in the iCT-Nav group and 42 patients in the FS group (Table 1). In the iCT-Nav group, four male patients (26.7%) and 11 female patients (73.3%) were included, and the median age was 59.3 years old (range: 27-76 years). The FS group included 42 patients with a median age of 45 years old (range: 20-61 years) younger than the iCT-Nav group, and 24 were females (57%). Both groups had similar BMI distribution. 

**Table 1:** Demographics of patients included in the study. *P-value is significant. iCT-Nav: intraoperative CT-guided navigation; FS: conventional fluoroscopy

		iCT-Nav (no. 15)	FS (no. 42)	p-Value
Age (year)		Median = 59.3 (range: 27-76)	Median = 45 (range: 20-61)	0.043*
Sex	Male	4 (26.7%)	18 (42.9%)	0.164
	Female	11 (73.7%)	24 (57.1%)	
BMI		29.5 (range: 23-34)	30.1 (range: 22-36)	0.765

Screw placement accuracy

**Figure 2:**
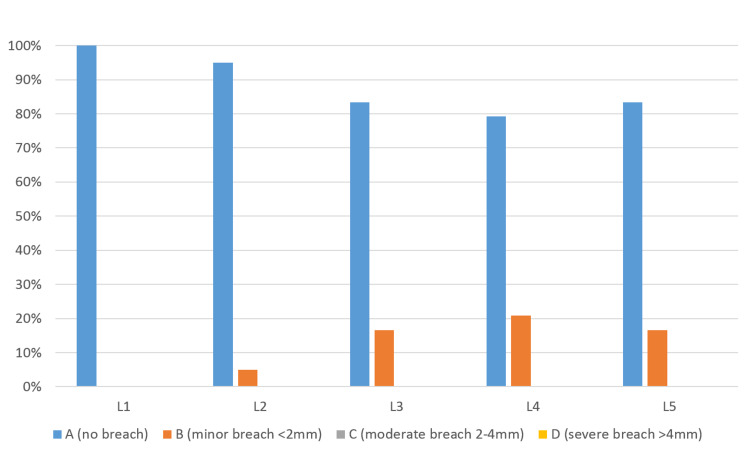
Intraoperative CT-guided navigation group, Gertzbein-Robbins classification of screw breach at lumbar spine, stratified by vertebrae level (L1 to L5).

Ninety-eight screws were inserted into the lumbar pedicles under an intraoperative CT-guided navigation system. No patients required intraoperative readjustment after the confirmation CT imaging. As per Gertzbein and Robbins classification (grade A), the number of screws that were optimally within the pedicle in iCT-Nav group was 85 screws (87.8%). Only 13 screws were placed less than 2 mm of the pedicular cortex (grade B) (Figure 2). 

**Figure 3:**
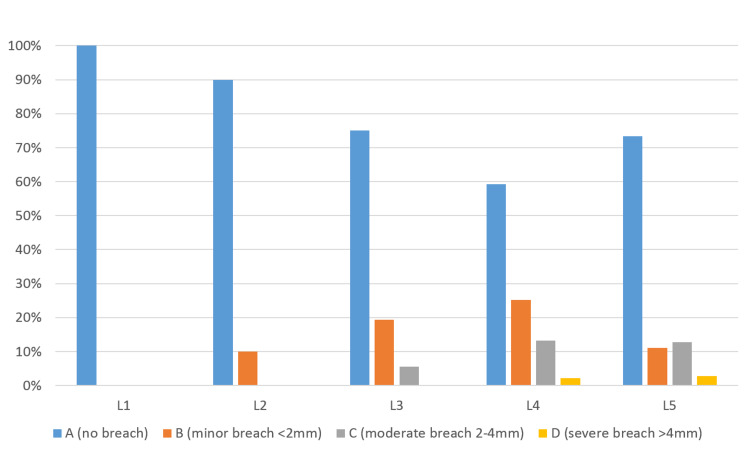
Conventional fluoroscopy group, Gertzbein-Robbins classification of screw breach at lumbar spine, stratified by vertebrae level (L1 to L5).

The most instrumented pedicles were L4 and L5 (n=12, 80%), while L3 were instrumented in nine patients (60%), L2 in 10 patients (66.7%), and L1 in six patients (40%) (Table 2). While in the FS group, the total number of screws inserted were 252 and distributed among each vertebral level as follows: L1 6 screws (2.3%), L2 10 screws (3.9%), L3 36 screws (14.3%), L4 91 screws (36.1%), and L5 109 screws (43.2%) (Figure 3). The number of optimal screw placement (grade A) was 176 screws (69.8%). The number of screws breaching the cortex (B-D) was 76 screws (30.1%).

**Table 2:** Summary of the results of pedicular screws placement in the iCT-Nav group. iCT-Nav: intraoperative CT-guided navigation

Vertebral level	No. of inserted screws	Total no. of breached screws	Minor <2 mm, (grade B)	Moderate 2-4 mm, (grade C)	Severe >4 mm, (grade D)
L1	12	0	0	0	0
L2	20	1 (5%)	1	0	0
L3	18	3 (16.6)	3	0	0
L4	24	5 (20.8)	5	0	0
L5	24	4 (16.6)	4	0	0
Total	98	13	13	0	0

Out of the 76 screws that have violated the cortex, 43 screws (56.57%) were grade B which is still considered safe placement. However, grade C screws were found to be 28 screws (36.8%), and only five screws (6.5%) were found to breach the cortex by more than 4 mm (grade D). The number of screws that were laterally deviated was 42 screws (55.3%), and those with medial perforation were 34 screws (44.7%). There were no cases with superior or inferior pedicular displacement (Table 3). 

**Table 3:** Summary of the results of pedicular screws placement in the FS group. FS: conventional fluoroscopy

Vertebral level	No. of inserted screws	No. of breached screws (grades B-D)	Medial breach	Lateral breach	Minor <2 mm, (grade B)	Moderate 2-4 mm, (grade C)	Severe >4 mm, (grade D)
L1	6	0	0	0	0	0	0
L2	10	1 (10%)	1 (100%)	0	1 (100%)	0	0
L3	36	9 (25%)	6 (66.7%)	3 (33.3%)	7 (77.8%)	2 (22.2%)	0
L4	91	37 (40.6%)	19 (51.4%)	18 (48.6%)	23 (62.2%)	12 (32.4%)	2 (5.4%)
L5	109	29 (26.6)	8 (27.6%)	21 (72.4%)	12 (41.4%)	14 (28.3%)	3 (10.3%)
Total	252	76	34	42	42	28	5

Based on moderate-severe screws displacement >2 mm (grades C and D) measured on computed tomography images, zero encountered displacement in the iCT-Nav group. In contrast, 33 displaced screws were identified in the FS group (p = 0.0001) (Table 4).

**Table 4:** Surgical outcomes and accuracy of screws placement. *P-value is significant. iCT-Nav: intraoperative CT-guided navigation; FS: conventional fluoroscopy; VAS: visual analog scale; SF-12 PCS: short 12 physical component scores; Preop: preoperative; M: month

		ICT-Nav group (N=15)	FS group (N=42)	p-Value
Mean (SD) hospital length of stay (days)		7.45(±2.5)	9.31(±2.05)	0.033*
VAS	Preop	7.34 (±1.27)	6.48(±0.82)	0.245
	3-M	2.10(±0.78)	3.78(±1.98)	0.043*
	2-year	0.65(±0.6)	2.36(±1.78)	0.024*
SF-12 PCS	Preop	27.16(±8.43)	26.67(±11.60)	0.349
	3-M	34.71(±10.50)	29.45(±14.40)	036*
	2-year	44.37(±8.74)	38.78(±12.87)	0.015*
No. of inserted pedicle screws		98	252	
L1		12	6	
L2		20	10	
L3		18	36	
L4		24	91	
L5		24	109	
No. of breached pedicles >2 mm (grades C and D)		0	33	0.0001*

Outcome and complications

Overall, the hospitalization time was shorter, and the functional outcome, particularly at two-year follow-up, was better in the iCT-Nav than FS group (Table 4). In addition, there were no cases of developed surgical site infection or cauda equina injury. 

Reoperation

No screws had to be repositioned intraoperatively in the CT/Nav group. However, in the FS group, two patients developed new symptoms postoperatively related to screw misplacement, requiring an extended length of stay and pain service consultation. One 70-year-old male had a new-onset moderate radicular pain after surgery due to L5 medially displaced screw, which partially improved with analgesic medication. However, he was readmitted a few weeks after surgery with worsening leg pain that required screw revision. The other patient was a 43-year-old female who developed new-onset severe leg pain, and weakness due to L4 medially displaced screw. She needed immediate surgical correction of screw position in less than 24 hours, and her leg pain improved, but she had a persistent weakness (foot drop of power 3/5) at a one-year follow-up.

## Discussion

Pedicular screw placement is a widely used technique for various clinical indications [2,3]. However, this procedure carries a risk of screw malposition, which can lead to serious neurological complications. Despite the small risk of neurological injury, severe neurological complications were reported [2,6]. In addition to neurological impairment such as new radicular pain, weakness or sensory loss, other damages such as vascular injuries, CSF leak, and reduction in the stability of the construct, all are other reported complications of misplaced screws [4,6].

Since the introduction of pedicle screws, placement accuracy has been the subject of many studies, in which a wide range of screw malposition rates (0.9-39.8%) has been reported [2-4,7,8]. A meta-analysis of studies aimed at assessing the accuracy of pedicle screw placement has found an overall rate of screw misplacement of 8.7%. In the subgroup of lumbar pedicle screw inserted in vivo, the median accuracy was 96.1% and 79% with and without navigation assistance, respectively [9]. In a recent meta-analysis for current techniques for placement of pedicle screws in the spine, a computed tomography image-guided navigation showed a statistically higher pedicle screw placement accuracy 95.5% compared with 91.5% for fluoroscopy assistance and 90.5% of robotic aid (p = 0.01 and 0.04, respectively), and a trend toward greater accuracy over freehand technique 93.1% (p = 0.11) [10]. It is interesting to note that a significant variation exists in the reporting of screw placement accuracy. This could be partly explained due to the difference in assessment methods, including the definition of displacement. Parker et al. reported an extensive series of thoracic and lumbar spine screws. They reported a low rate of lumbar screws malposition (0.9%) in 3100 screws placed in the lumbar spine [3]. We believe this low rate is because they only reported screws that had breached the cortex by more than 25%. Many other studies have included even minor minimal breaches of the cortex. Most screws violate the pedicular cortex by less than 4 mm, causing no neurological symptoms [11,12]. The same finding is reported in our series, with only 6.5% of misplaced screws that have breached the cortex by more than 4 mm in the FS group. None have been reported in the iCT-Nav group. 

Also, the learning curve for surgeons in using the different modalities for pedicle screw insertion impacts the accuracy outcome. Rivkin and Yocom reported a significant increase in accuracy rates between the beginning and the end of their experience with 1651 pedicle screws placed in 266 patients. Suboptimal accuracy (82.9%) was observed for the first 15 patients, whereas it increased to 92.6% and 95.9% for the subsequent patients (p < 0.05) [13]. Fichtner et al. elucidated the effect of the learning curve in their series for the revision rate of misplaced pedicle screws of the thoracolumbar spine comparing 3D fluoroscopy navigated (3D-FL) with freehand technique, which decreased in the 3D-FL group from 0.9% in 2011 to 0.3% in 2015 [14].

Farber et al. were the first to study the accuracy of pedicular screw placement using plain X-rays radiographs and CT scans. They studied 16 consecutive patients who underwent pedicular screw instrumentation. They found that CT scans have shown significantly more pedicular screws violations, especially medially (10 times more accurate), than expected compared to plain X-ray radiography [7]. Medial violation of pedicular screws seems to be more dangerous due to the risk of neurological damage. Gertzbien and Robbins were the first to postulate the idea of the "safe zone" of pedicular screws malposition. They found that less than 2 mm violation of the pedicular canal is considered safe with a low risk of neurological damage [15]. Their postulated grading system based on 2 mm increments seems to be the most widely accepted method for determining pedicle screw placement accuracy [15]. We have used their system to report our data. Many studies have shown that the risk of neurological damage is low, with violations less than 4 mm [4,5]. In our series, the two only reported patients with neurological deficits secondary to screw malposition had screws that were medially misplaced by more than 4 mm. Most breaches in our series resulted from lateral malposition (55.3%), in agreement with many other studies [2,3,16]. This may be attributed to the thicker medial cortical wall of the pedicle, as well as the desire of the surgeon to avoid potential injury to the spinal cord and cauda equina [3].

The incidence of neurological symptoms secondary to nerve root irritation by a misplaced screw is rare, ranging between 0.6% and 11% [6]. New postoperative pain or a neurological deficit requires a careful evaluation of postoperative images to correlate with the clinical picture. In most cases, a new neurological symptom secondary to a screw misplacement necessitates a surgical revision [6].

The emergence of new intraoperative navigation systems has significantly increased the accuracy of pedicle screw placement. Many reviews have demonstrated an increase in efficiency when navigation systems are used [9,12,15,17-19]. Tian et al. have provided a systematic review of articles studying pedicle screw insertion accuracy with or without the assistance of image-guided systems. They found that CT-based navigation, a 3D fluoroscopy-based navigation system, and 2D fluoroscopy-based navigation systems (as used in our cases) were associated with statistically significantly less pedicle violation incidence than traditional freehand methods [12]. Besides, the use of intraoperative CT-based and 3D fluoroscopy-based navigation systems provided more accurate pedicle screw insertion over the 2D fluoroscopy-based navigation system (the same fluoroscopy we used in our patients) [12]. The superiority of screw placement accuracy using intraoperative CT-based or three-dimension fluoroscopy-based navigation systems has been shown in an extensive systematic analysis [19], as well in a large retrospective study by Fichtner et al. [14].

Moreover, Kosmopoulos and Schizas reported a meta-analysis of 130 studies of pedicle screw placement accuracy. They found that the median pedicle screw placement accuracy in the lumbar spine in the navigation-assisted subgroup (96.1%) was higher than that of the subset without the use of navigation (79.0%) [9,20]. However, there was no statistically significant difference in lumbar pedicle screw accuracy between O-arm (Siemens AG, Munich, Germany) and iCT-AIRO systems among the different navigation systems (Farah et al., 90.8% 92.2%, respectively) [21]. The same findings were reported by Scarone et al. with a 99.2% accuracy rate in iCT-AIRO and 99.7% in the O-arm group [22].

In our study, the accuracy rate of lumbar pedicle screw placement (Gertzbein and Robbins grades A and B) is 100% when using intraoperative CT-guided navigation and 86.9% when using conventional fluoroscopy, with a statistically significant difference (p = 0.0001) between both methods. These rates seem to be acceptable when comparing them with accuracy rates reported in the literature.

The intraoperative navigation systems are not without disadvantages. Besides their effect on prolonging operative time, other obstacles include increasing the equipment density in the operating room during surgery, equipment pollution, and increasing the risk of exposure to radiation during operation [20,23]. Also, procedures may be prolonged further due to technical limitations as calibration errors, occasional blocking of the camera field of view, and inadvertently touching/hitting reference frames [6]. In addition to all of that, many hospitals, especially in developing countries, do not have the luxury of having an intraoperative navigation system [23]. 

It can be assumed that more accurate screw placement using navigation systems should result in better patient outcomes [15]. Verma et al. have done a systematic review and tried to address the increased accuracy in screw placement through navigation systems, leading to a significant decrease in complication rates from the misplacement of pedicle screws. They found that despite the statistically significant increase in accuracy, screws placed through navigation systems do not show statistically significant benefits in reducing neurological complications [17]. There was insufficient data in the literature to infer a conclusion regarding fusion rate, pain relief, and health outcome scores when comparing navigation over conventional pedicle screw insertion [17]. 

Our study carries certain limitations that should be mentioned. First, it’s a retrospective analysis of prospectively collected data. Second, the sample size is relatively small and not randomized. Additionally, we did not include any description of pedicle screw diameter, width or trajectory, or whether the bicortical purchase was obtained. Nonetheless, the early results of our work seem to corroborate other studies, and more comprehensive follow-up studies with larger sample sizes are needed.

## Conclusions

We demonstrated that the use of intraoperative CT-guided navigation could be associated with improved reliability and accuracy of pedicle screws placement in degenerative lumbar spine surgery. Our study's screw placement accuracy was translated with an associated shorter hospital stay, better outcomes, no readmission, and revision surgery. In addition, iCT-guided navigation is a safe adjunct to spinal surgery. We believe it is cost-effective and decreases surgeons and operating room staffs radiation exposure. Additional large prospective randomized studies and research can be done to further evaluate its accuracy and reliability.
